# The impact of perioperative anemia on postoperative delirium after hip fracture surgeries in older adults: a multicenter retrospective cohort study

**DOI:** 10.3389/fnut.2025.1683080

**Published:** 2026-01-15

**Authors:** Xin Xu, Peng Xu, Junxiang Wang, Hui Yu, Yanan Li, Qi Zhang, Tao Wang, Yubin Long, Erliang Li, Jiachen Wang, Jiale Xie, Junfei Guo

**Affiliations:** 1 Department of Joint Surgery, Honghui Hospital, Xi’an Jiaotong University, Xi’an, Shanxi, China; 2 Xi'an Key Laboratory of Pathogenesis and Precision Treatment of Arthritis, Xi’an, Shanxi, China; 3 Department of Anesthesiology, Hebei Medical University Third Hospital, Shijiazhuang, Hebei, China; 4 Department of Lower Limb Trauma, Beijing Jishuitan Hospital, Guizhou Hospital, Guiyang, Guizhou, China; 5 Department of Orthopaedics Surgery, Baoding First Central Hospital, Baoding, Hebei, China

**Keywords:** postoperative delirium, anemia, hip fracture surgery, older adults, multicenter, retrospective cohort study

## Abstract

**Background:**

Postoperative delirium (POD) is a frequent yet underestimated neurocognitive complication following hip fracture surgery, particularly among older adults. While anemia is a prevalent and potentially modifiable nutritional disorder in this population, but its impact on POD remains uncertain. We aimed to examine the association between perioperative anemia and POD risk in older adults undergoing surgical treatment for intertrochanteric fractures (ITF).

**Methods:**

We conducted a multicenter retrospective cohort study of elderly patients with ITF who underwent surgical fixation in China. Patients were categorized by their nadir perioperative hemoglobin (Hb): ≥103 g/L, 75–<103 g/L, and <75 g/L. Propensity score matching (PSM) with a 1:1:1 optimal algorithm was used to balance baseline variables. Participants were followed for up to 24 months. POD incidence was assessed and compared across groups. We used multivariable logistic regression to identify independent predictors and performed exploratory mediation analyses.

**Results:**

Among 1,694 patients, the overall POD incidence was 9.7%. After PSM, POD incidence differed across Hb groups: 12.4% (<75 g/L), 2.9% (75–<103 g/L), and 7.6% (≥103 g/L). Multivariate analyses identified severe anemia (OR: 3.13; 95% CI: 1.59–6.18; *p* < 0.001), preoperative transfusions (OR: 1.67, 95% CI: 1.02–2.74, *p* = 0.042), and transfusion volume (OR: 1.39, 95% CI: 1.02–1.89, *p* = 0.037) as independent predictors of POD. Exploratory mediation analyses showed the lowest perioperative Hb levels accounted for 55.9% of the effect of anemia on POD, with preoperative transfusions and transfusion volume accounting for 35.4 and 8.7%, respectively.

**Conclusion:**

Severe perioperative anemia is strongly associated with increased POD risk in older adults with ITF, independent of other clinical factors. Optimizing perioperative anemia management should be a central component of surgical care and POD prevention strategies in geriatric orthopedic populations.

## 1 Introduction

Hip fractures are increasingly common in aging populations and demand substantial surgical and perioperative resources (1, 2). Intertrochanteric fractures (ITF) account for a large proportion of orthopedic admissions and impose a considerable public health burden due to multimorbidity and associated socioeconomic costs (3, 4). Despite improvements in caring for these vulnerable individuals, the one-year mortality rates are alarmingly high, with percentages ranging from 7 to 10% within a month and 12 to 35% within the initial year (5, 6). Notably, in-hospital mortality may approach 10% even with optimal management (7).

As a prevalent and significant complication following hip fracture surgery, postoperative delirium (POD) is an acute brain dysfunction characterized by impaired consciousness and altered cognitive functioning (8–11). Approximately 23% of hospitalized patients experience POD, which is associated with prolonged length of stay, higher healthcare expenditures, and increased readmissions and mortality (12, 13). Neuman et al. (14) reported an alarming 36% mortality rate at six-month follow-up in patients who developed POD, with related medicare expenditures exceeding 6.5 billion dollars annually (15). Numerous studies have identified various risk factors for delirium, including advanced age, body mass index (BMI), American Society of Anesthesiologists (ASA) classification, malnutrition, general anesthesia, parkinsonism, polypharmacy, anemia, depression, dementia, cognitive decline, frailty, alcohol use, visual or hearing impairment, and institutionalization (16–20).

However, some of these risk factors remain controversial, and controlling symptoms through medication alone often does not yield satisfactory outcomes (21, 22). Against this backdrop, anemia merits focused attention as a common and potentially modifiable preoperative condition. Pathophysiologically, anemia may exacerbate cerebral hypoxia and organ vulnerability, contributing to adverse neurological outcomes (23–25). Inadequately managed anemia in hip fracture patients has been linked to functional decline, longer hospitalization, and worse morbidity and mortality (26–29). Observational data suggest that moderate-to-severe anemia is an independent risk factor for POD (30), whereas erythrocyte transfusion, though frequently used, may not prevent POD and could itself be associated with delirium risk (31–33). These findings are particularly relevant in older adults, in whom multimorbidity and malnutrition are common (34). Nevertheless, despite emerging evidence, the link between anemia and delirium remains inadequately explored and subject to debate (35, 36).

To address this gap, we conducted a multicenter retrospective cohort study across four specialist trauma centers at large tertiary referral university hospitals to examine the association between perioperative anemia and POD in older adults undergoing surgical treatment for ITF. Our objective was to generate clinically actionable evidence to inform risk stratification and guide interventions for POD in geriatric orthopedic populations.

## 2 Methods

### 2.1 Study design, setting, and population

**Figure 1:**
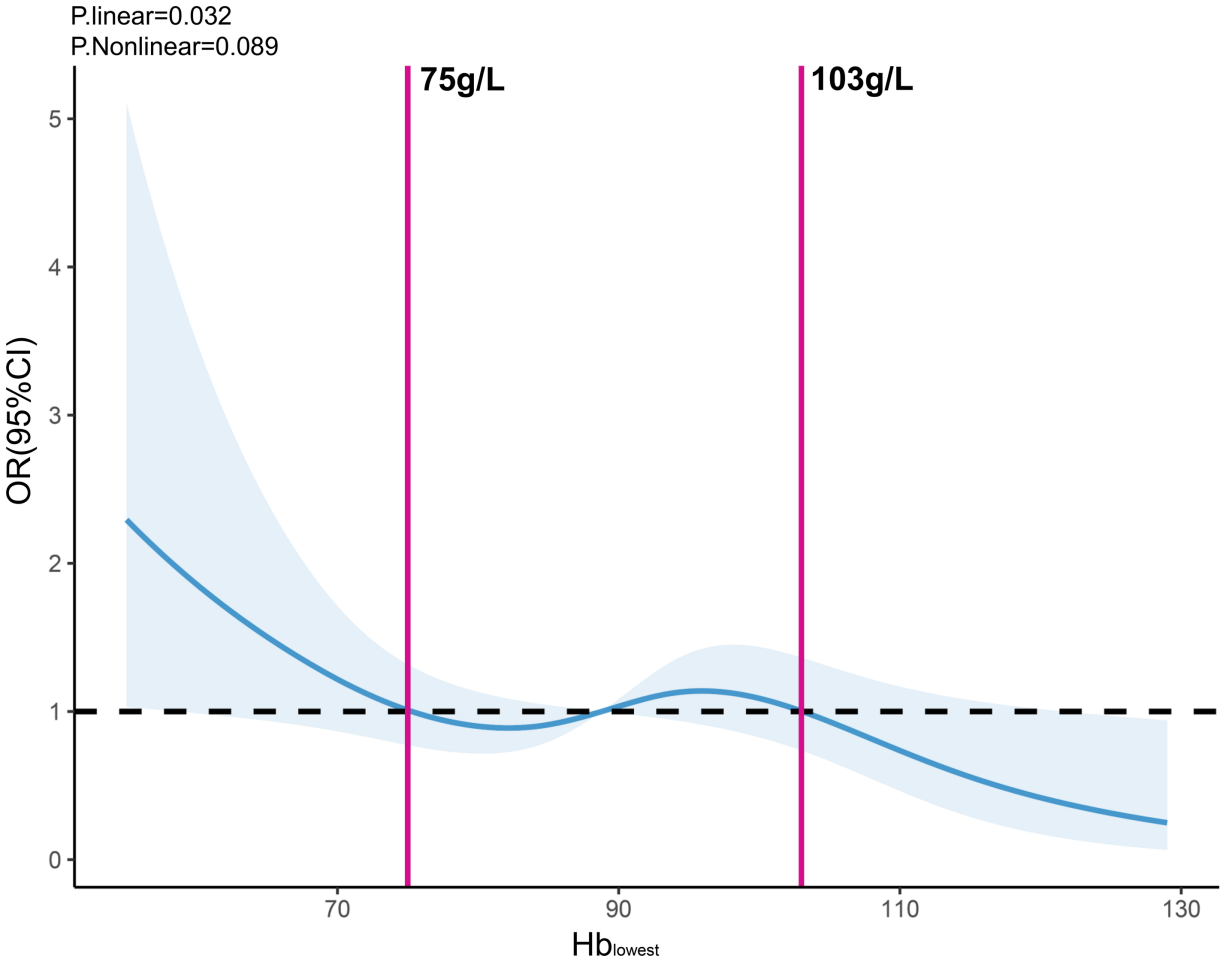
Dose-effect relationship between the lowest perioperative Hb level and POD. Evaluated by using a restricted cubic spline model with four knots and adjusted by all perioperative covariables with *p*-value <0.1. *p*-nonlinearity showed no statistical significance, which was estimated using the likelihood ratio test comparing the restricted cubic spline model with the linear model. Relative risks were indicated by blue solid line and 95% CIs by blue area, in which the reference points indicated by pink solid lines were 75 g/L and 103 g/L for the Hb_lowest_. POD, postoperative delirium; Hb_lowest_, the lowest perioperative hemoglobin level.

This multicenter retrospective study included all ITF patients undergoing intramedullary fixation with the proximal femoral nail anti-rotation (PFNA) and was conducted in four large Level I trauma centers located in tertiary referral university hospitals in China between July 2022 and June 2023. All centers, located in major urban areas, adhered to standardized treatment protocols (32). Inclusion criteria included individuals who were 65 years or older, with an admission delay of less than 48 h from initial injury, and were followed up for at least 2 years. Patients who had open or pathological fractures, additional fractures of the ITF, multiple injuries, inability to communicate, mental illness, refusal of surgery, and those treated conservatively due to severe comorbidities were excluded based on specific criteria. Patients were categorized into three groups based on the lowest perioperative hemoglobin (Hb) level: Hb ≥ 103 g/L, 103 > Hb ≥ 75 g/L, and Hb < 75 g/L. The reference points of 75 and 103 g/L were determined using the restricted cubic spline (RCS) curve, which evaluates linear and nonlinear associations in the dose–response relationship between the lowest perioperative Hb level and POD (Figure 1).

As an observational study, clinical decision-making remained uninfluenced by investigators. Clinical information during the perioperative period was gathered through predetermined forms, with regular follow-ups at 1, 3, 6, 12, and 24 months after surgery through phone conversations or outpatient appointments. The study protocol was approved by Ethics Committees of the four participating institutions of Honghui Hospital, Xi’an Jiaotong University, Hebei Medical University Third Hospital, Beijing Jishuitan Hospital, Guizhou Hospital, and Baoding First Central Hospital in accordance with the principles of the Declaration of Helsinki (as revised in 2013). All participants or their immediate family members provided written informed consent. To protect privacy, patient information was de-identified. This study adhered to the guidelines outlined in the Helsinki Declaration and the findings were presented in accordance with the Strengthening the Reporting of cohort, cross-sectional, and case–control studies in Surgery (STROCSS) criteria (37).

### 2.2 Assessment of postoperative delirium

POD was diagnosed according to DSM-5 criteria, based on daily bedside evaluations performed by trained clinicians from postoperative day 1 until discharge. Evaluations systematically assessed acute onset and fluctuation, inattention, and disturbances in awareness and cognition, and excluded alternative explanations (e.g., coma, intoxication). A single evaluation meeting full DSM-5 criteria was considered diagnostic of POD. Clinicians received standardized training before study start, and uncertain cases were resolved by senior adjudication.

### 2.3 Covariates and definitions

In order to reduce potential confounding bias, a wide variety of possible confounding variables were taken into account using both current research and expert knowledge. Data were extracted from electronic patient records at both institutions, including sex, age, BMI, residence (rural or urban), smoking or drinking, the commonly used visual analog scores (VAS), Geriatric Depression Scale (GDS), functional independence measure (FIM), presence of anxiety, the ASA grade (classified as I to VI), modified Elixhauser comorbidity method (mECM) (38), fracture type, time from injury to hospital admission/surgery, anesthesia type, intraoperative blood loss, surgical duration, and transfusion volume.

Analyses were conducted on the outcomes related to complications such as POD, sudden death, acute heart failure, acute respiratory failure, myocardial infarction, cerebral peduncles, stress ulcer, arrhythmia, pulmonary infection, electrolyte disbalance, hypoproteinemia, stress hyperglycemia, and deep vein thrombosis (DVT), as well as the average LOS and survival time. During the follow-up period, information on survival status and date of death was collected. Follow-up started when the cohort was enrolled, with the endpoint being either death from any cause or the latest follow-up visit, whichever occurred first. Subsequently, mortality rates were documented at 30-day, 90-day, 180-day, 12-month, and 24-month intervals.

**Figure 2:**
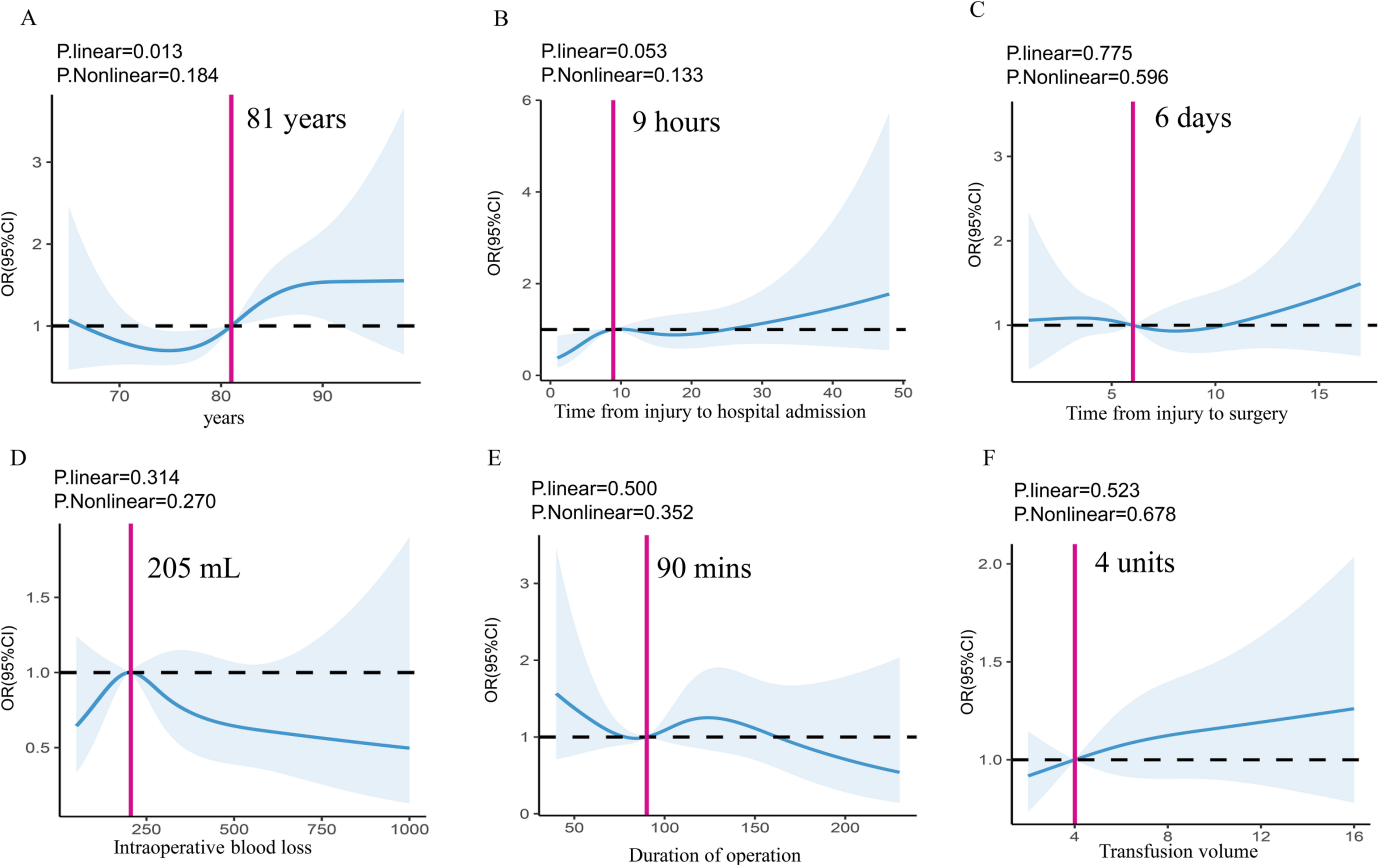
Dose-effect relationship between patient age **(A)**, time from injury to hospital admission **(B)**, time from injury to surgery **(C)**, intraoperative blood loss **(D)**, duration of operation **(E)**, transfusion volume **(F)**, and POD. Evaluated by using a restricted cubic spline model with four knots and adjusted by all perioperative co-variables with *p*-value <0.1. *p*-nonlinearity showed no statistical significance, which was estimated using the likelihood ratio test comparing the restricted cubic spline model with the linear model. Relative risks were indicated by blue solid line and 95% CIs by blue area, in which the reference points indicated by pink solid lines were 81 years, 9 h, 6 days, 205 mL, 90 min, and 4 units for the patient age **(A)**, time from injury to hospital admission **(B)**, time from injury to surgery **(C)**, intraoperative blood loss **(D)**, duration of operation **(E)**, and transfusion volume **(F)**. POD, postoperative delirium.

BMI classifications were defined as follows: normal (<24 kg/m^2^), overweight (24 ≤ BMI < 28 kg/m^2^), and obesity (≥28 kg/m^2^). Upon admission, the mECM evaluated the comorbidities of patients, categorizing them into groups of less than 0, 0, 1 to 5, 6 to 13, and 14 or more. The 15-question GDS and FIM assessed symptoms of depression and the capacity to carry out ADL, respectively (2). Hb levels at admission were categorized based on thresholds 8 g/dL, 10 g/dL, and 12 g/dL. We followed the standard clinical guidelines (39–41) for perioperative anemia management in elderly hip fracture patients. As for blood transfusions, we adhered to the current restrictive guidelines when the Hb value was less than 80 g/L in general patients or between 80 and 100 g/L with a serious cardiac condition or when anemia becomes symptomatics, which is general practice in hospitals (42, 43). In addition, transfusions were performed preoperatively, intraoperatively, and postoperatively, depending on the patient’s condition and intraoperative blood loss. RCS curves set reference points for patient age, time from injury to hospital admission, time from injury to surgery, intraoperative blood loss, duration of operation, and transfusion volume at specific values of 81 years, 9 h, 6 days, 205 mL, 90 min, and 4 units, respectively (Figure 2).

### 2.4 Statistical analysis

To evaluate the normality of continuous variables, the Shapiro–Wilk test was utilized. Categorical data was presented in terms of frequency and percentage, whereas continuous data was presented as the mean and standard deviation (SD). Continuous and categorical variables were compared using Student’s t-tests and chi-square tests, respectively. Missing data for continuous BMI (2.3%) were imputed using linear regression. To account for potential linear and nonlinear associations in the dose–response relationships, RCS with four nodes were used for controlling for all covariates with a significance level of less than 0.1, which were placed at the 5th, 35th, 65th, and 90th percentiles of the distribution for continuous variables based on best practices and recommendations from statistical literature, suggesting that positioning the knots at these percentiles provides an optimal balance between flexibility and stability of the model (44). The choice of four knots, as opposed to a higher or lower number, was guided by both the size of our dataset and the need for sufficient flexibility without introducing excessive complexity into the model. Empirical research demonstrates that models with four to five knots are generally appropriate for datasets of moderate size, such as ours, allowing for the detection of nonlinear patterns without reducing statistical power (45–47). The analyses visually examined the dose-effect relationship between the lowest perioperative Hb level, age, time from injury to hospital admission, time from injury to surgery, intraoperative blood loss, duration of operation, transfusion volume, and POD, setting reference points based on RCS shapes and stratifying related data into subgroups accordingly.

To enhance the reliability of our findings and guarantee their applicability to actual clinical settings, we employed propensity score matching (PSM) with a 1:1:1 optimal matching algorithm via the caliper matching of 0.05 to address differences in baseline characteristics across the three groups, ultimately minimizing the effects of selection bias and potential confounding variables. Besides, our study primarily assessed the lowest perioperative hemoglobin levels as a determinant of POD and we acknowledge blood transfusions are commonly administered to correct significant perioperative anemia and the subsequent changes in hemoglobin levels could impact perioperative transfusions and then impact postoperative outcomes, including the risk of POD, particularly for patients who received substantial transfusions. Therefore, we additionally conducted univariate logistical and multiple regression analyses and then mediation analysis was carried out by using multivariate regression analyses, to determine relationships and analyze the mediating effect. The mediation effects were assessed using the product of coefficients method, which directly assessed whether the mediation or indirect effect was significant. In the multivariate analysis, we grouped perioperative transfusion into six categories: whether received preoperative transfusion, intraoperative transfusion, and postoperative transfusion or not, and the total preoperative, intraoperative, and postoperative transfusion volume. Dichotomous outcomes were reported using the odds ratio (OR) and 95% CI. All data analyses were performed using IBM SPSS Statistics for Windows, version 26.0 (IBM, Armonk, NY, USA). A two-tailed *p* value <0.05 was considered significant.

## 3 Results

### 3.1 Patient characteristics and group comparisons

Between July 2022 and June 2023, 2,899 consecutive patients with ITF were screened for eligibility, and 1,694 were included in the final analysis after applying exclusion criteria. In total, 1,205 patients were eliminated based on the exclusion criteria. In particular, there were 303 patients younger than 65, 162 who were treated conservatively, 514 with a delay in admission of 48 h or longer, 87 with open hip fractures, pathological fractures, or multiple injuries, 62 who were unable to communicate or had mental illness, and 77 who were lost to follow-up. The average age of participants was 80.2 years (range 65–106), with an overall POD incidence of 9.7% (145/1694). Baseline characteristics across the three Hb groups (Hb ≥ 103 g/L, 103 > Hb ≥ 75 g/L, and Hb < 75 g/L) differed significantly before PSM in variables such as age, BMI, and transfusion volume. After PSM, all groups showed comparable demographic and clinical characteristics, with the exception of transfusion volume (all *p* > 0.05, Table 1).

**Table 1:** Comparisons of baseline characteristics before and after matching in three groups of participants.

Characteristics	Before Matching				After Matching			
	Hb≥103 g/L (*N* = 301)	103 > Hb≥75 g/L (*N* = 1,163)	Hb<75 g/L (*N* = 230)	*p* value	Hb≥103 g/L (*N* = 210)	103 > Hb≥75 g/L (*N* = 210)	Hb<75 g/L (*N* = 210)	*p* value
Sex				0.002*				0.079
Men	114 (37.9%)	323 (27.8%)	61 (26.5%)		75 (35.7%)	59 (28.1%)	55 (26.2%)	
Women	187 (62.1%)	840 (72.2%)	169 (73.5%)		135 (64.3%)	151 (71.9%)	155 (73.8%)	
Age group				0.007*				0.587
<81 years	175 (58.1%)	561 (48.2%)	110 (47.8%)		108 (51.4%)	100 (47.6%)	110 (52.4%)	
≥81 years	126 (41.9%)	602 (51.8%)	120 (52.2%)		102 (48.6%)	110 (52.4%)	100 (47.6%)	
BMI group				0.006*				0.084
Normal	175 (58.1%)	794 (68.3%)	156 (66.4%)		126 (60.0%)	139 (66.2%)	141 (67.1%)	
Overweight	99 (32.9%)	283 (24.3%)	64 (27.8%)		67 (31.9%)	50 (23.8%)	60 (28.6%)	
Obesity	27 (9.0%)	86 (7.4%)	10 (4.3%)		17 (8.1%)	21 (10.0%)	9 (4.3%)	
Residence				0.502				0.153
Rural	97 (32.2%)	414 (35.6%)	77 (33.5%)		70 (33.3%)	87 (41.4%)	71 (33.8%)	
Urban	204 (67.8%)	749 (64.4%)	153 (66.5%)		140 (66.7%)	123 (58.6%)	139 (66.2%)	
Smoking				0.075				0.382
No	210 (69.8%)	830 (71.4%)	148 (64.3%)		122 (58.1%)	135 (64.3%)	141 (67.1%)	
Yes	91 (30.2%)	333 (28.6%)	82 (35.7%)		88 (41.9%)	75 (35.7%)	69 (32.9%)	
Drinking				<0.001*				0.185
No	296 (98.3%)	1,118 (96.1%)	206 (89.6%)		205 (97.6%)	203 (96.7%)	198 (94.3%)	
Yes	5 (1.7%)	45 (3.9%)	24 (10.4%)		5 (2.4%)	7 (3.3%)	12 (5.7%)	
VAS	5.06 (1.79)	5.35 (1.78)	5.20 (1.63)	0.117	4.94 (1.80)	5.27 (1.87)	5.19 (1.65)	0.142
GDS	4.16 (1.42)	4.11 (1.46)	4.12 (1.44)	0.219	4.14 (1.43)	4.11 (1.27)	4.13 (1.41)	0.984
FIM				0.641				0.837
No	150 (49.8%)	611 (52.5%)	116 (50.4%)		111 (52.9%)	105 (50.0%)	107 (51.0%)	
Yes	151 (50.2%)	552 (47.5%)	114 (49.6%)		99 (47.1%)	105 (50.0%)	103 (49.0%)	
Anxiety				0.954				0.503
No	246 (81.7%)	942 (81.0%)	186 (80.9%)		174 (82.9%)	179 (85.2%)	170 (81.0%)	
Yes	55 (18.3%)	221 (19.0%)	44 (19.1%)		36 (17.1%)	31 (14.8%)	40 (19.0%)	
ASA				0.146				0.875
1	47 (15.6%)	209 (18.0%)	54 (23.5%)		34 (16.2%)	41 (19.5%)	47 (22.4%)	
2	97 (32.2%)	313 (26.9%)	66 (28.7%)		68 (32.4%)	65 (31.0%)	65 (31.0%)	
3	106 (35.2%)	445 (38.3%)	71 (30.9%)		74 (35.2%)	66 (31.4%)	64 (30.5%)	
4	46 (15.3%)	161 (13.8%)	33 (14.3%)		29 (13.8%)	30 (14.3%)	28 (13.3%)	
5	5 (1.7%)	35 (3.0%)	6 (2.6%)		5 (2.4%)	8 (3.8%)	6 (2.9%)	
mECM				0.641				0.460
<0	5 (1.7%)	26 (2.2%)	2 (0.9%)		3 (1.4%)	5 (2.4%)	2 (1.0%)	
0	144 (47.8%)	566 (48.7%)	127 (55.2%)		108 (51.4%)	96 (45.7%)	117 (55.7%)	
1–5	56 (18.6%)	194 (16.7%)	34 (14.8%)		36 (17.1%)	35 (16.7%)	32 (15.2%)	
6–13	82 (27.2%)	321 (27.6%)	55 (23.9%)		56 (26.7%)	59 (28.1%)	49 (23.3%)	
≥14	14 (4.7%)	56 (4.8%)	12 (5.2%)		7 (3.3%)	15 (7.1%)	10 (4.8%)	
Fracture type				0.018*				0.921
Stable	178 (59.1%)	592 (50.9%)	111 (48.3%)		104 (49.5%)	101 (48.1%)	100 (47.6%)	
Unstable	123 (40.9%)	571 (49.1%)	119 (51.7%)		106 (50.5%)	109 (51.9%)	110 (52.4%)	
Time from injury to hospital admission				0.500				0.875
≥9 h	156 (51.8%)	627 (53.9%)	115 (50.0%)		109 (51.9%)	105 (50.0%)	104 (49.5%)	
<9 h	145 (48.2%)	536 (46.1%)	115 (50.0%)		101 (48.1%)	105 (50.0%)	106 (50.5%)	
Time from injury to surgery				0.679				0.835
≥6 days	152 (50.5%)	606 (52.1%)	125 (54.3%)		114 (54.3%)	116 (55.2%)	120 (57.1%)	
<6 days	149 (49.5%)	557 (47.9%)	105 (45.7%)		96 (45.7%)	94 (44.8%)	90 (42.9%)	
Type of anesthesia				0.744				0.826
General anesthesia	111 (36.9%)	454 (39.0%)	86 (37.4%)		75 (35.7%)	77 (36.7%)	81 (38.6%)	
Regional anesthesia	190 (63.1%)	709 (61.0%)	144 (62.6%)		135 (64.3%)	133 (63.3%)	129 (61.4%)	
Intraoperative blood loss				0.744				0.618
≥205 mL	124 (41.2%)	491 (42.2%)	91 (39.6%)		87 (41.4%)	88 (41.9%)	79 (37.6%)	
<205 mL	177 (58.8%)	672 (57.8%)	139 (60.4%)		123 (58.6%)	122 (58.1%)	131 (62.4%)	
Duration of operation				0.289				0.207
≥90 min	200 (66.4%)	775 (66.6%)	141 (61.3%)		144 (68.6%)	132 (62.9%)	127 (60.5%)	
<90 min	101 (33.6%)	388 (33.4%)	89 (38.7%)		66 (31.4%)	78 (37.1%)	83 (39.5%)	
Transfusion volume				<0.001*				<0.001*
<4 units	57 (18.9%)	805 (69.2%)	218 (94.8%)		37 (17.6%)	114 (54.3%)	198 (94.3%)	
≥4 units	244 (81.1%)	358 (30.8%)	12 (5.2%)		173 (82.4%)	96 (45.7%)	12 (5.7%)	

Values are presented as the number (%) or mean (SD).

BMI, body mass index; VAS, visual analog scores; GDS, Geriatric Depression Scale; FIM, functional independence measure; ASA, American Society of Anesthesiologists; mECM, modified Elixhauser’s Comorbidity Measure.

* *p* < 0.05, statistical significance.

### 3.2 Incidence of postoperative delirium (POD) and other outcomes

The incidence of POD varied significantly across Hb groups after PSM: Hb < 75 g/L (12.4%), 103 > Hb ≥ 75 g/L (2.9%), and Hb ≥ 103 g/L (7.6%). Statistical analyses revealed a significant association between severe anemia (Hb < 75 g/L) and POD (*p* = 0.001). Table 2 provides a detailed overview of pre- and post-matching results, which encompass mortality rates, complications, mean LOS, and mean survival time.

**Table 2:** Comparisons of mortalities, complications, LOS, and survival time before and after matching in three groups of patients.

Characteristics	Before matching				After matching			
	Hb≥103 g/L (*N* = 301)	103 > Hb≥75 g/L (*N* = 1,163)	Hb<75 g/L (*N* = 230)	*p* value	Hb≥103 g/L (*N* = 210)	103 > Hb≥75 g/L (*N* = 210)	Hb<75 g/L (*N* = 210)	*p* value
One-month mortality (Yes)	3 (1.0%)	9 (0.8%)	4 (1.7%)	0.441	2 (1.0%)	2 (1.0%)	4 (1.9%)	0.620
Three-month mortality (Yes)	6 (2.0%)	18 (1.5%)	5 (2.2%)	0.742	5 (2.4%)	3 (1.4%)	5 (2.4%)	0.716
Six-month mortality (Yes)	10 (3.3%)	43 (3.7%)	18 (7.8%)	0.012*	6 (2.9%)	5 (2.4%)	15 (7.1%)	0.026*
One-year mortality (Yes)	22 (7.3%)	83 (7.1%)	30 (13.0%)	0.009*	15 (7.1%)	13 (6.2%)	29 (13.8%)	0.012*
Two-year mortality (Yes)	41 (13.6%)	169 (14.5%)	58 (25.2%)	<0.001*	30 (14.3%)	32 (15.2%)	52 (24.8%)	0.009*
POD (Yes)	21 (7.0%)	96 (8.3%)	28 (12.2%)	0.085	16 (7.6%)	6 (2.9%)	26 (12.4%)	0.001*
Sudden death (Yes)	1 (0.3%)	8 (0.7%)	2 (0.9%)	0.686	0 (0.0%)	1 (0.5%)	2 (1.0%)	0.249
Acute heart failure (Yes)	22 (7.3%)	98 (8.4%)	28 (12.2%)	0.115	17 (8.1%)	20 (9.5%)	22 (10.5%)	0.701
Acute respiratory failure (Yes)	8 (2.7%)	29 (2.5%)	7 (3.0%)	0.889	6 (2.9%)	4 (1.9%)	7 (3.3%)	0.655
Myocardial infarction (Yes)	3 (1.0%)	3 (0.3%)	2 (0.9%)	0.191	3 (1.4%)	3 (1.4%)	1 (0.5%)	0.513
Cerebral peduncles (Yes)	7 (2.3%)	44 (3.8%)	5 (2.2%)	0.265	2 (1.0%)	6 (2.9%)	5 (2.4%)	0.319
Stress ulcer (Yes)	2 (0.7%)	23 (2.0%)	9 (3.9%)	0.029*	2 (1.0%)	1 (0.5%)	7 (3.3%)	0.049*
Arrhythmia (Yes)	58 (19.3%)	206 (17.7%)	38 (16.5%)	0.703	44 (21.0%)	42 (20.0%)	34 (16.2%)	0.421
Pulmonary infection (Yes)	23 (7.6%)	143 (12.3%)	23 (10.0%)	0.061	16 (7.6%)	26 (12.4%)	20 (9.5%)	0.257
Electrolyte disbalance (Yes)	139 (46.2%)	557 (47.9%)	109 (47.4%)	0.868	105 (50.0%)	91 (43.3%)	96 (45.7%)	0.381
Hypoproteinemia (Yes)	96 (31.9%)	349 (30.0%)	76 (33.0%)	0.591	74 (35.2%)	54 (25.7%)	71 (33.8%)	0.077
Stress hyperglycemia (Yes)	84 (27.9%)	294 (25.3%)	60 (26.1%)	0.648	56 (26.7%)	47 (22.4%)	57 (27.1%)	0.467
DVT				0.676				0.456
No	177 (58.8%)	633 (54.4%)	122 (53.0%)		120 (57.1%)	108 (51.4%)	112 (53.3%)	
Intermuscular vein and peroneal vein thrombosis	96 (31.9%)	412 (35.4%)	78 (33.9%)		71 (33.8%)	78 (37.1%)	72 (34.3%)	
Posterior tibial vein thrombosis	11 (3.7%)	49 (4.2%)	11 (4.8%)		9 (4.3%)	13 (6.2%)	9 (4.3%)	
Popliteal vein thrombosis	10 (3.3%)	25 (2.1%)	8 (3.5%)		7 (3.3%)	4 (1.9%)	8 (3.8%)	
Superficial and deep femoral vein thrombosis	3 (1.0%)	15 (1.3%)	5 (2.2%)		2 (1.0%)	1 (0.5%)	4 (1.9%)	
Common femoral vein thrombosis	4 (1.3%)	29 (2.5%)	6 (2.6%)		1 (0.5%)	6 (2.9%)	5 (2.4%)	
Mean LOS	14.65 (5.44)	14.59 (6.83)	16.14 (8.35)	0.006*	14.89 (5.69)	14.79 (6.95)	16.04 (8.16)	0.127
Mean survive time	52.23 (26.74)	46.02 (25.20)	46.09 (26.59)	0.057	53.05 (27.34)	45.53 (25.31)	45.36 (26.15)	0.003*

Values are presented as the number (%) or mean (SD).

POD, postoperative delirium; DVT, deep vein thrombosis, LOS, length of hospital stay.

* *p* < 0.05, statistical significance.

Statistical analysis showed a significant difference in mean LOS among the three groups before PSM, but this discrepancy became insignificant after PSM. Significantly, the disparities in POD occurrence and mean survival time became notable following the matching process (*p* = 0.001 and *p* = 0.003, respectively), in contrast to their lack of significance before matching. Significant differences in 6-month, 1-year, and 2-year mortality rates and stress ulcer incidence were also observed among groups post-PSM, (all *p* < 0.05). There was no significant difference in complication or mortality rates at 30- and 90-day intervals before or after PSM.

### 3.3 Multivariate analysis and mediation analysis

**Figure 3:**
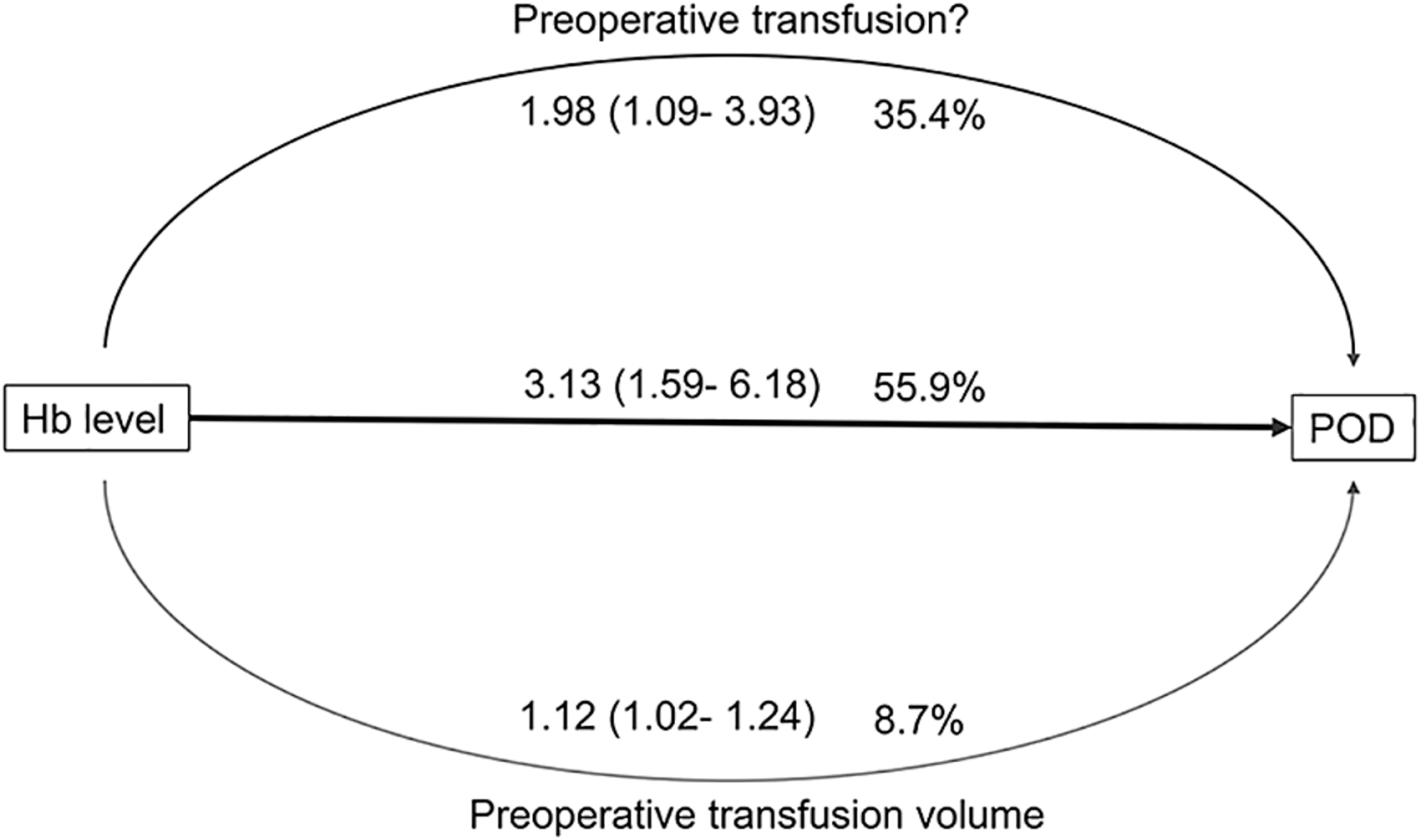
Mediation analysis results of anemia and delirium influenced by whether preoperative transfusion and total preoperative transfusion volume. POD, postoperative delirium; Hb_lowest:_ the lowest perioperative hemoglobin level.

Multivariate logistic regression analysis identified severe anemia (OR: 3.13, 95% CI: 1.59–6.18, *p* < 0.001), preoperative transfusions (OR: 1.67, 95% CI: 1.02–2.74, *p* = 0.042), and total preoperative transfusion volume (OR: 1.39, 95% CI: 1.02–1.89, *p* = 0.037) as significant predictors of POD. Mediation analysis showed that 55.9% (OR: 3.13, 95% CI: 1.59–6.18) of the total effect of anemia on POD was mediated by the lowest perioperative Hb levels, while preoperative transfusions and transfusion volume accounted for 35.4% (OR: 1.98, 95% CI: 1.09–3.93) and 8.7% (OR: 1.12, 95% CI: 1.02–1.24), respectively (Table 3; Figure 3).

**Table 3:** The univariate and multivariate analyses of all involved possible factors associated with POD.

Characteristics	Univariate				Multivariate	*p* value
	Total (*n* = 1,694)	No POD (*n* = 1,549)	POD (*n* = 145)	*p* value	OR (95% CI)	
Sex				0.002*		
Men	498 (29.4%)	439 (28.3%)	59 (40.7%)		Reference	
Women	1,196 (70.6%)	1,110 (71.7%)	86 (59.3%)		0.45 (0.19, 1.07)	0.070
Age group				0.015*		
<81 years	846 (49.9%)	788 (50.9%)	58 (40.0%)		Reference	
≥81 years	848 (50.1%)	761 (49.1%)	87 (60.0%)		3.75 (1.67, 8.44)	0.001*
BMI group				0.628		
Normal	1,125 (66.4%)	1,028 (66.4%)	97 (66.9%)			
Overweight	446 (26.3%)	411 (26.5%)	35 (24.1%)			
Obesity	123 (7.3%)	110 (7.1%)	13 (9.0%)			
Residence				0.583		
Rural	577 (34.1%)	531 (34.3%)	46 (31.7%)			
Urban	1,117 (65.9%)	1,018 (65.7%)	99 (68.3%)			
Smoking				0.040*	3.46 (1.19, 10.09)	0.023*
Never	1,238 (73.1%)	1,145 (73.9%)	93 (64.1%)			
Past	98 (5.8%)	87 (5.6%)	11 (7.6%)			
Current	358 (21.1%)	317 (20.5%)	41 (28.3%)			
Drinking				<0.001*		
No	1,630 (96.2%)	1,525 (98.5%)	105 (72.4%)		Reference	
Yes	64 (3.8%)	24 (1.5%)	40 (27.6%)		5.43 (1.98, 14.88)	<0.001*
VAS	5.28 (1.76)	5.23 (1.80)	5.80 (1.33)	<0.001*	2.11 (1,04, 4.27)	0.038*
GDS	4.12 (1.44)	3.94 (1.29)	6.11 (1.51)	<0.001*	1.97 (1.32, 2.95)	<0.001*
FIM				<0.001*		
No	877 (51.8%)	849 (54.8%)	28 (19.3%)		Reference	
Yes	817 (48.2%)	700 (45.2%)	117 (80.7%)		2.77 (1.49, 5.15)	0.013*
Anxiety				<0.001*		
No	1,374 (81.1%)	1,303 (84.1%)	71 (49.0%)		Reference	
Yes	320 (18.9%)	246 (15.9%)	74 (51.0%)		1.69 (0.72, 3.96)	0.229
The lowest perioperative Hb level				0.085**	3.13 (1.59, 6.18)	<0.001*
Hb ≥ 103 g/L	301 (17.8%)	280 (18.1%)	21 (14.5%)			
103 > Hb ≥ 75 g/L	1,163 (68.7%)	1,067 (68.9%)	96 (66.2%)			
Hb < 75 g/L	230 (13.6%)	202 (13.0%)	28 (19.3%)			
ASA				<0.001*	2.63 (0.68, 10.20)	0.162
1	310 (18.3%)	294 (19.0%)	16 (11.0%)			
2	476 (28.1%)	448 (28.9%)	28 (19.3%)			
3	622 (36.7%)	561 (36.2%)	61 (42.1%)			
4	240 (14.2%)	207 (13.4%)	33 (22.8%)			
5	46 (2.7%)	39 (2.5%)	7 (4.8%)			
mECM				0.279		
<0	33 (1.9%)	32 (2.1%)	1 (0.7%)			
0	837 (49.4%)	765 (49.4%)	72 (49.7%)			
1–5	284 (16.8%)	252 (16.3%)	32 (22.1%)			
6–13	458 (27.0%)	423 (27.3%)	35 (24.1%)			
≥14	82 (4.9%)	77 (5.0%)	5 (3.4%)			
Fracture type				0.862		
Stable	881 (52.0%)	807 (52.1%)	74 (51.0%)			
Unstable	813 (48.0%)	742 (47.9%)	71 (49.0%)			
Time from injury to hospital admission				0.298		
≥9 h	898 (53.0%)	815 (52.6%)	83 (57.2%)			
<9 h	796 (47.0%)	734 (47.4%)	62 (42.8%)			
Time from injury to surgery				0.665		
≥6 days	883 (52.1%)	810 (52.3%)	73 (50.3%)			
<6 days	811 (47.9%)	739 (47.7%)	72 (49.7%)			
Type of anesthesia				0.284		
General anesthesia	651 (38.4%)	589 (38.0%)	62 (42.8%)			
Regional anesthesia	1,043 (61.6%)	960 (62.0%)	1,043 (61.6%)			
Intraoperative blood loss				0.113		
≥205 mL	706 (41.7%)	655 (42.3%)	51 (35.2%)			
<205 mL	988 (58.3%)	894 (57.7%)	94 (64.8%)			
Duration of operation				0.714		
≥90 min	1,116 (65.9%)	1,018 (65.7%)	98 (67.6%)			
<90 min	578 (34.1%)	531 (34.3%)	47 (32.4%)			
Total transfusion volume group				0.470		
<4 units	1,080 (63.8%)	983 (63.5%)	97 (66.9%)			
≥4 units	614 (36.2%)	566 (36.5%)	48 (33.1%)			
Total transfusion volume	4 (2, 6)	4 (2, 6)	4 (2, 6)	0.246		
Preoperative transfusion				0.023*		
No	1,094 (64.6%)	1,013 (65.4%)	81 (55.9%)		Reference	
Yes	600 (35.4%)	536 (34.6%)	64 (44.1%)		1.67 (1.02, 2.74)	0.042*
Intraoperative transfusion				0.223		
No	529 (31.2%)	477 (30.8%)	52 (35.9%)			
Yes	1,165 (68.8%)	1,072 (69.2%)	93 (64.1%)			
Postoperative transfusion				0.224		
No	811 (47.9%)	749 (48.4%)	62 (42.8%)			
Yes	883 (52.1%)	800 (51.6%)	83 (57.2%)			
Preoperative transfusion volume	0 (0, 2)	0 (0, 2)	0 (0, 2)	0.041*	1.39 (1.02, 1.89)	0.037*
Intraoperative transfusion volume	2 (0, 2)	2 (0, 2)	2 (0, 2)	0.256		
Postoperative transfusion volume	2 (0, 2)	2 (0, 2)	2 (0, 2)	0.225		

Values are presented as the number (%) or mean (SD) or median (IQR).

BMI, body mass index; VAS, visual analog scores; GDS, Geriatric Depression Scale; FIM, functional independence measure; ASA, American Society of Anesthesiologists; mECM, modified Elixhauser’s Comorbidity Measure.

*p < 0.05, statistical significance.

**p < 0.10, statistical significance.

## 4 Discussion

To the best of our knowledge, POD is not only a frequent occurrence following hip surgery but a significant cause of postoperative mortality. Alarmingly, clinicians fail to recognize and address POD in up to 80% of cases, highlighting a critical gap in postoperative care (48). Previous studies have reported that the incidence of POD in older individuals varies from 2 to 7% in Asian countries, with rates exceeding 50% following hip fractures and cardiac surgeries (49, 50). Therefore, assessing and mitigating risk factors for POD in hospitalized patients is of utmost importance. Choi et al. (49) demonstrated that risk factors such as cognitive decline, parkinsonism, and higher ASA scores are linked to a higher likelihood of developing POD after hemiarthroplasty. Additionally, a comprehensive meta-analysis (19) found a strong association between cognitive dysfunction, BMI, albumin concentrations, and various underlying health conditions with POD following hip fracture surgery. Despite numerous studies attempting to identify key risk factors influencing POD, findings remain inconsistent and controversial. Recent research indicates that hematological indices, including the administration of red blood cell (RBC) transfusions, may also play a role in the progression or mitigation of POD (17, 32, 51). This suggests a potential, yet unexplored, relationship between anemia and POD.

Previous studies examining the link between anemia and POD have yielded conflicting results. For instance, a study of surgical elderly patients found no significant association between anemia and delirium, attributing this to low statistical power (35). Conversely, a cohort study with a larger sample size identified a significant independent association between moderate-to-severe anemia and delirium (30). More recently, a prospective cohort analysis concluded that postoperative anemia is highly prevalent and increases the risk of POD as well as prolongs the LOS (29). These inconsistencies highlight the need for further investigation into the relationship between anemia and POD, considering the limitations of previous studies such as small sample sizes and uncontrolled confounding factors.

In this study, we utilized a multicenter retrospective cohort design to explore the potential link between perioperative anemia and POD. Our cohort study findings demonstrated that ITF patients with severe anemia (lowest perioperative Hb level <75 g/L) are more susceptible to POD. The biological mechanisms underlying the relationship between anemia and POD may involve the body’s dependence on hemoglobin for oxygen uptake and transport. Anemic states, characterized by low Hb levels and leads to a reduction in oxygen-carrying capacity, which can result in chronic or acute hypoxia, ultimately leading to brain dysfunction, cognitive impairment, and mood abnormalities (52–54). Importantly, the brain is highly sensitive to oxygen deprivation, and hypoxia has been shown to disrupt normal neuronal function, contributing to cognitive impairment and neuroinflammation—both of which are known precursors to delirium. Besides, hypoxia can also lead to increased oxidative stress and mitochondrial dysfunction, triggering neuroinflammatory responses which can impair normal brain metabolism and further exacerbate cognitive decline and contribute to delirium (55–57). Brombo et al. (30) found no overall association was discovered between anemia and delirium, however, a significant correlation was noted when anemia was combined with cognitive decline, suggesting that anemia-induced delirium may be mediated by cognitive functions. Specifically, anemia in middle-aged and elderly populations is often accompanied by malnutrition and chronic diseases, particularly in elderly patients with pre-existing vulnerabilities such as frailty or comorbidities (34, 58).

In addition, anemia-related hypoxia can impair cerebral blood flow and the delivery of essential nutrients to the brain. The brain may adapt to chronic anemia through mechanisms such as modulating cervical oxygen partial pressure receptors and cerebral blood flow (59). Hoffman et al. (60) found that the brain response to hypoxia was diminished in older rats compared to younger rats, suggesting that elderly patients’ ability to adapt to anemia may mask a potential link between anemia and POD. Conversely, another study indicated that elderly patients with reduced cerebral oxygenation are more prone to developing delirium postoperatively, especially when combined with the physiological stress of surgery (29). We speculate that substantial blood loss during hip fracture surgeries may expose elderly patients to acute anemia, which their brains have not yet adapted to, resulting in POD and negatively impacting clinical outcomes and mortality. Moreover, Weiskopf et al. found that acute anemia, characterized by a sharp drop in Hb concentration, was associated with decreased responsiveness, impairing cognitive function and memory in humans (61). Furthermore, the co-occurrence of anemia and delirium is often seen in elderly populations where malnutrition and chronic disease further compromise brain function, increasing the susceptibility to POD.

In summary, the risk of POD may be related to the severity of anemia and the patient’s ability to tolerate it. This causal inference may also be interpreted by our retrospective cohort study findings, which suggest that the Hb < 75 g/L group has the highest frequency of POD (12.4%), while the 103 > Hb ≥ 75 g/L group has the lowest frequency of POD (2.9%). Interestingly, the Hb ≥ 103 g/L group had the second-highest rate of POD (7.6%), approximately 2.6 times higher than the 103 > Hb ≥ 75 g/L group. The high frequency of extensive blood transfusions (≥4 units) in the Hb ≥ 103 g/L group could explain our previous research results (17, 32). These complex results may explain the contradictions among previous observational studies. Importantly, anemia, being an easily assessed and modifiable indicator, is simple and feasible for predicting and managing POD. However, it is crucial to consider whether the patient has adapted to chronic anemia. Future studies with larger sample sizes are necessary to investigate the correlation between various types of symptomatic anemia and POD.

This study has several strengths. First, in a multicenter retrospective cohort we adjusted for numerous previously identified risk factors as covariates, enhancing the robustness of the findings. Second, we complemented the multicenter observational cohort study with mediation analyses, thereby bolstering the credibility and reliability of our conclusions. Nonetheless, several limitations warrant consideration. First, we did not have complete data on anesthetic and perioperative medications and therefore could not adjust for their use. Certain agents are known to influence delirium risk (e.g., ketamine, opioids, and benzodiazepines), whereas others are not (e.g., dexmedetomidine and lidocaine). Lack of control for these exposures may have introduced residual confounding. Second, the number of POD events was modest, which limited the statistical power for additional stratified or interaction analyses. Third, our cohort was intentionally restricted to a homogeneous surgical technique with the same internal fixation. Although this design choice reduces procedural heterogeneity and the attendant risk of bias, it may limit generalizability because different operative approaches can differentially affect delirium risk. Fourth, despite the strength of use of PSM to control for confounding variables, certain variables remained unbalanced after matching. This residual imbalance is a known limitation of PSM, particularly in large, heterogeneous cohorts where subtle differences between subgroups may persist. While PSM improves comparability between groups, it cannot entirely eliminate all discrepancies, especially in observational studies with complex datasets. It is important to acknowledge that unaccounted-for variables such as unmeasured perioperative variables, other protocols for treating anemia including iron supplementation both oral or intravenous, erythropoiesis-stimulating agents and oxygen therapy may still influence the results, and further studies utilizing alternative methods may be needed to validate these findings. Another notable limitation of our study is the exclusion of patients with pre-existing mental illness and those unable to communicate may introduce selection bias, which may reduce the generalizability of our findings to real-world clinical settings. Specifically, older adults with cognitive impairments or psychiatric comorbidities may present unique risk factors for both anemia and POD that were not captured in our study. Consequently, caution is warranted when applying our conclusions to broader clinical populations, particularly those more vulnerable to POD due to mental health or neurological conditions. This would ensure a more comprehensive understanding of how anemia and categorize patients based on the etiology of anemia (e.g., iron deficiency anemia, anemia of chronic disease, vitamin B12 deficiency) and examine the distinct pathways through which each type of anemia contributes to POD across diverse patient populations, thereby enhancing the clinical applicability of the findings in future research. Lastly, considering the total effect of exposure can pass both through the mediators (indirect effect) and through a direct pathway (direct effect), the role of the mediating effect cannot be overstated. However, the inclusion of important covariates as potential confounders in the mediation analyses ensures that estimates of the mediation effects are less likely to be exaggerated. In conclusion, while PSM was employed to reduce confounding, it cannot completely eliminate all residual bias inherent in observational studies. To mitigate this, we complemented the observational study with mediation analyses, which strengthens the causal inference and minimizes the risk of confounding, thereby providing a more reliable estimate of the impact of perioperative anemia on POD. Consequently, caution is warranted when applying our conclusions to broader clinical populations, particularly those more vulnerable to POD due to mental health or neurological conditions. This would ensure a more comprehensive understanding of how anemia and categorize patients based on the etiology of anemia (e.g., iron deficiency anemia, anemia of chronic disease, vitamin B12 deficiency) and examine the distinct pathways through which each type of anemia contributes to POD across diverse patient populations, thereby enhancing the clinical applicability of the findings in future research.

## 5 Conclusion

In this multicenter retrospective cohort of older adults undergoing surgery for ITF, severe perioperative anemia was strongly associated with an increased risk of postoperative delirium, independent of other clinical factors. This research provides a significant step forward in understanding the importance of optimizing perioperative anemia management as a key component of surgical nutritional care and POD prevention strategies in geriatric orthopedic populations. Given the observational design, prospective validation is warranted. Future research should include multicenter prospective cohorts and adequately powered randomized trials to evaluate structured anemia-management strategies using standardized delirium assessments and comprehensive measurement of perioperative exposures.

## Data Availability

The original contributions presented in the study are included in the article/supplementary material, further inquiries can be directed to the corresponding authors.
